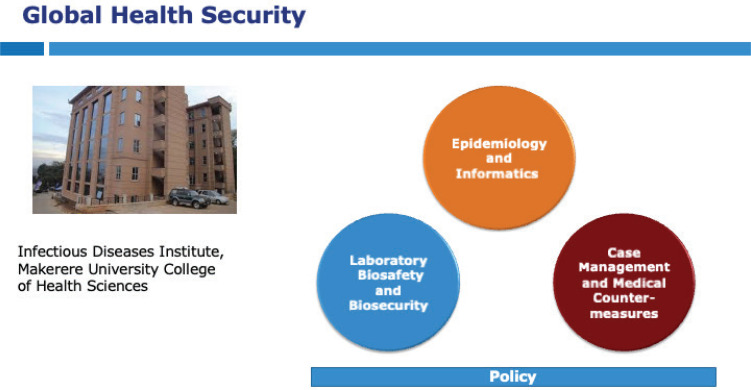# Support for the response to COVID-19 in Uganda: contribution of the global health security program at Makerere University's Infectious Diseases Institute

**DOI:** 10.4314/ahs.v22i2.13S

**Published:** 2022-08

**Authors:** Mohammed Lamorde, Rodgers Ayebare, Daniel Bulwadda, Judith Nanyondo, Lydia Nakiire, Richard Walwema, Morgan Otita, Peter Mukiibi, Immaculate Nabukenya, Francis Kakooza, Andrew Kambugu

**Affiliations:** Infectious Diseases Institute, Makerere University. P. O. Box 22418, Kampala, Uganda

**Keywords:** Epidemics, University, Ebola

## Abstract

**Background:**

Outbreaks are occurring at increasing frequency and they require multisectoral and multi-stakeholder involvement for optimal response. The Global Health Security Agenda is a framework that governments and other stakeholders can use to strengthen countries' capacities to prevent, detect and respond to outbreaks but there are few examples of academic programs using this approach.

**Methods:**

This is a narrative review of contributions of Makerere University through the Global Health Security Program at the Infectious Diseases Institute (IDI). Information was sourced from peer-reviewed publications and grey literature highlighting work done between 2017 – 2021.

**Results:**

Aligned to GHSA, IDI made contributions to strengthen national and subnational capacities for biosafety and biosecurity, sample collection and transportation, electronic disease surveillance, infection prevention and control, case management prior to COVID-19 that were subsequently used to support response efforts for COVID-19 in Uganda.

**Conclusion:**

The IDI Global Health Security program provides a model that can be used by institutions to deliberately develop capacities relevant to outbreak preparedness and response.

## Introduction

National governments have the responsibility and the mandate to protect the health of their citizens. For infectious disease outbreaks, there is not only risk for spread and illness within countries but a potential for spread to other countries and territories that result in a mutual obligation to act prevent spread domestically and to share information promptly with the international community when there is potential for international spread or when high impact events occur. The World Health Organization (WHO) International Health Regulations 2005 (IHR) provides the international framework that defines capacities that member states require to manage outbreaks and other public health events^1^. Since 2019, public health actions have focused on COVID-19 response, while bringing attention to the state of emergency preparedness for future disease outbreaks. The discovery of a novel coronavirus (SARS-CoV 2 virus) unveiled the scenario of a previously unknown pathogen, transmitted efficiently via the respiratory route, with potential to cause severe illness and death on an unprecedented scale^2^.

While investigations into the origins of the virus and initial steps taken since its discovery continue, the critical question is whether countries are better prepared for the next potential pandemic. Are systems in place to prevent, rapidly detect and effectively respond to the outbreak in a manner that prevents another pandemic? The Global Health Security Preparedness Index, which measures preparedness capacities, found similar scores for many countries in 2019 and 2021, suggesting that advancement in these capacities is not the inevitable consequence of the experience gained while managing a pandemic^3^. Instead, specific, systematic steps are neede with support from multiple players to rapidly achieve progress in preparedness. Through a voluntary country peer review mechanism known as the Joint External Evaluation, countries identify gaps in capacities that are used to develop national action plans for health security^4^. Over the last decade, specific strategies have been enacted to support countries to close gaps in preparedness and response capacities^5^. The Global Health Security Agenda is an effort by nations, international organizations, and civil society to accelerate progress towards a world that is safe and secure from infectious disease threats by prioritizing global health security and by establishing capacity to prevent, detect and rapidly respond to biological threats^6^. Countries are encouraged to fully implement global frameworks including IHR and the World Organization for Animal Health Performance of Veterinary Services pathway^6^,^7^. An important and element of GHSA was inclusion of non-state partners including academic organizations, civil society organizations and the private sector to support health security activities. This report highlights how capacities developed by the Global Health Security program of the Infectious Diseases Institute (IDI) Makerere University were leveraged to contribute to the COVID-19 response in Uganda.

## Methods

In 2018, IDI created a dedicated Global Health Security program to support country capabilities to prevent, detect and respond to infectious disease outbreaks and other biological threats. This is a narrative review summarizing information from peer-reviewed manuscripts and conference abstracts and grey literature (IDI strategic plan (8), and IDI annual reports available at https://idi.mak.ac.ug/resources/annual-reports/) highlighting work done by IDI relevant to outbreaks). Manuscript searches were conducted in an online database (https://pubmed.ncbi.nlm.nih.gov) using IDI author names (e.g Lamorde M) for papers published between 01 Jan 2017 and 31 December 2021.

## Results/Discussion

We identified the IDI Strategic Plan and the following annual reports (2016–2017, 2017–2018, 2019–2020 and 2020–2021) and reviewed content related to the Global Health Security program at IDI. Additionally, using author searches 5 peer reviewed manuscripts were identified relevant to the work of the GHS program.

## Capacity building aligned to the Global Health Security Agenda

The IDI GHS program aims to support health systems in Africa to develop capabilities to prevent, detect and respond to infectious disease outbreaks and biological threats. Three core areas were identified for action, first, to strengthen national laboratory systems for detection of pathogens capable of causing outbreaks and to enhance biosafety and biosecurity Second, to enhance data systems for surveillance and reporting of outbreaks and prevention of emergence and spread of antimicrobial resistance using a One Health approach. Third, to enhance infection prevention control, case management and medical countermeasures during outbreaks.

IDI uses the Potter and Brough Capacity Pyramid model to assess gaps at five levels including cognizance of local context (eg. local policies, strategies, cultural and religious values). Identifying structures and systems (e.g defining roles and responsibilities of stakeholders and relevant organizations), provision of staff and infrastructure; developing skills necessary for executing tasks and provision of tools to enable activities or duties^8^,^9^. Across projects the same approach was used to identify gaps in a systematic manner for capacity development.

An important feature of IDI programming is personnel integration across technical areas, rather than treating individual technical areas as silos, so the same personnel responsible for biosafety and biosecurity activities would also implement work in national laboratory systems for detecting outbreaks or antimicrobial resistance. In addition, staff in selected technical areas would also engage in outbreak response by embedding within government teams leading the effort and providing complementary support in infection prevention and control, laboratory, surveillance and border health, as required. These core technical areas were underpinned by a policy team working with government to advance and monitor progress during the implementation of the Uganda National Action Plan for Health Security. Initial capacity building activities were focused on viral hemorrhagic fever syndromes since outbreaks of Ebola virus disease (Ebola), Marburg virus disease, rift valley fever and Crimean-Congo hemorrhagic fever have all been reported in Uganda 10,11

## Strengthened systems for biosafety and biosecurity and laboratory detection of pathogens

The program scaled microbiology capabilities of eight laboratories by providing equipment and supplies and training 35 laboratory staff to detect outbreaks using real-time polymerase chain reaction techniques^12^. IDI supported harmonization of the bio risk management curriculum and trained 25 national auditors^12^. IDI also supported the registration and launch of the Biosafety and Biosecurity Association of Uganda to create a network of human and veterinary experts in biosafety and biosecurity. A key activity supported was funding for the First Regional Biosecurity conference in Kampala on November 14–15, 2017 with support from US CDC and the Dutch National Institute for Public Health and the Environment^13^. The conference created awareness and generated discussion on policy and legislation, multi-sectoral collaboration, prevention of bio-terrorism and safe transfer of biological agents.

## Enhanced sample transportation and data systems for surveillance and reporting of outbreaks

The program conducted a successful pilot of the integration of animal samples into the national sample transport of results return system in human health. This allowed leveraging of investments in human health to allow separately packaged suspected outbreak samples collected from animals to be transported from peripheral health facilities and brought to regional veterinary laboratory laboratories and to the national reference laboratory for animal health (NADDEC). In 2017, systems for surveillance were strengthened by working with partners to support a pilot of trainings and mentorships of the electronic integrated disease surveillance and response (e-IDSR) system in 11 districts in West Nile. The eIDSR system consists three platforms; the cell phone messaging android and web-based that can enables data integration using a One Health approach^12^. Crucially, a One Health approach was used to ensure the functionality of the system was cascaded to health facilities, animal health workers and community health workers.

## Partnerships established for infection prevention control, case management and medical countermeasures

Uganda has experienced several outbreaks caused by Ebola and Marburg viruses. IDI joined the Joint Mobile Emerging Disease Intervention Clinical Capability (JME-DICC) consortium in collaboration with Makerere University Walter Reed Project (MUWRP) and United States Department of Defense partners to improve capacity for clinical research for investigational therapeutics for filoviruses with the aim of generating data leading to product licensure by developing: a) clinical capacity to identify and manage potential research participants through training of clinical staff; b) laboratory capacity through lab renovations, provision of equipment, and training of lab staff; and c) clinical research capability through renovations of an isolation research ward. These capacities were developed at the Fort Portal Regional Referral Hospital where an ongoing sepsis observational study is underway^10^,^11^.

Following an outbreak of Ebola in neighboring Democratic Republic of the Congo in 2018, assessments in Uganda revealed infection prevention and control gaps for key capacities in health facilities including screening, isolation and notification systems and capacity to manage suspected Ebola cases. IDI in collaboration with US CDC and other national partners, supported the development of an Infection Prevention and Control preparedness and response training for frontline health workers working in routine health settings and supported the training of the health workers to acquire competencies for routine screening, isolation and notification for suspect Ebola cases while maintaining high standards of IPC and to improve knowledge, skills in management of a suspect Ebola cases^14^.

Furthermore, with capacity previously established through JMEDICC, IDI also supported erection of semi-permanent isolation unit in Naguru China Uganda Friendship Hospital and renovation of Oli Health Centre IV isolation in Arua. Following these improvements, training and drills were conducted for 41 frontline staff working at Naguru for EVD suspected cases. In high risk districts, 49 clinicians were trained on case management and 40 laboratory staff were trained on laboratory protocols for Ebola12. Through the CAPA-CT II project, IDI conducted a clinical study on the first drug-drug interaction study between the antiretroviral drug tenofovir disoproxil fumarate and remdesivir (an unsuccessful candidate therapeutic for ebola that was subsequently approved for COVID-19)^15^.

## Leveraging capacities for COVID-19

In February 2020, prior to first case of COVID-19 reported in Uganda, IDI initiated its contribution for COVID-19 preparedness and the subsequent response. Internally, IDI adapted its business continuity plan for pandemic influenza to the COVID-19 pandemic. IDI embedded teams in the Ministry of Health to support coordination and planning in various response pillars and it supported development of legal instruments (rules) linked to the Public Health Act for COVID-19 prevention and control, development of capacity building materials, facilitated training of initial case management teams and supported border assessments and laboratory sampling capacity building targeting designated points of entry. With the detection of the earliest cases in Uganda, IDI supported quarantine, contact tracing and surveillance activities, and capacity building for COVID-19 laboratory sample management and reporting by mentoring 1371 laboratory workers in 500 health facilities and training health workers to activate sample collection in 53 points of entry^16^.

The COVID-19 pandemic placed health care workers (HCWs) at greater risk of infection and death. Among HCWs, COVID-19 exposures and infections result in work restriction or staff isolation, respectively, leading to loss of personnel needed to continue the delivery of essential health services. Adapting mentorship previously for Ebola, IDI supported the development of a national IPC mentorship program for COVID-19. IDI supported capacity building activities for IPC at regional level in five regions (Arua, Mubende, Kabarole, Mbale and Kabale regions) and implemented direct IPC mentorship at facility level in 27 districts in West Nile, Bunyoro, greater Buganda regions and Kampala, Wakiso, Mubende, Kabarole and Mbale districts. Within 6 months (March – September 2020), IDI supported intensive and subsequently, monthly mentorship visits reaching 1615 health facilities and 10,500 HCW^17^. Similar mentorship cascades were developed for daily electronic monitoring for COVID-19 laboratory testing and surveillance in 24 districts in Uganda (districts in West Nile, Central and Eastern Uganda health regions)^18^. Similarly, COVID-19 surveillance mentorships used a training cascade from regional, district, facility level and to community level in West Nile, Eastern Uganda, South West Uganda, and Rakai and Kyotera districts. During community transmission of COVID-19, IDI obtained support from the US CDC to provide technical support and facilitate government teams to support capacity building for emergency operations centers, emergency medical services, emergency response, vaccination and water sanitation and hygiene. In particular for WASH, IDI supported production of over 10,000 liters of alcohol-based hand rub in high-risk (mostly border) districts to allow equitable distribution to health facilities^19^.

## Conclusion

The IDI Global Health Security program provides a model that can be used by institutions to deliberately develop capacities relevant to outbreak preparedness and response. Technical collaborations and partnerships contributed to the success of this program.

## Figures and Tables

**Figure 1:**